# Predictive Neural Network Modeling of Nanoporous Anodic Alumina for Controlled Drug Release Implants: An Integrated Machine Learning Approach

**DOI:** 10.3390/ma19091705

**Published:** 2026-04-23

**Authors:** Ao Wang, Wan Fahmin Faiz Wan Ali, Muhamad Azizi Mat Yajid, Jianjun Gu

**Affiliations:** 1 Faculty of Mechanical Engineering, Universiti Teknologi Malaysia, Johor Bahru 81310, Malaysia; 2 College of Physics and Electronic Engineering, Hebei Minzu Normal University, Chengde 067000, China; 3 Hebei Province Chengde Center for Technological Innovation in Clean Energy and Ecological Protection, Chengde 067000, China

**Keywords:** nanoporous anodic alumina, drug-releasing implant, controlled release, neural network modeling, pore structure, diffusion, machine learning, artificial intelligence

## Abstract

Background: Nanoporous anodic alumina (NAA) has emerged as a promising platform for localized drug delivery in biomedical implants owing to its tunable nanoscale pore structure and biocompatibility. However, achieving the desired pore characteristics currently relies on time-consuming trial-and-error adjustments of anodization parameters. Methods: We developed a comprehensive data-driven machine learning framework using a feed-forward artificial neural network (ANN) with three hidden layers (64-32-16 neurons) trained on 77 samples from a compiled dataset of 99 anodization experiments spanning 1995–2025. The model predicts the NAA pore diameter based on anodization conditions (electrolyte type, concentration, voltage, temperature, and time). Results: The ANN achieved R^2^ = 0.803, root mean square error (RMSE) = 25.83 nm, and mean absolute error (MAE) = 17.05 nm on training data; however, 5-fold cross-validation revealed moderate generalization (CV R^2^ = 0.471 ± 0.078). Multiple linear regression showed comparable training performance (R^2^ = 0.804) but superior cross-validation (CV R^2^ = 0.729 ± 0.083). Feature importance analysis identified anodization voltage (29.15% ANN importance) and electrolyte type (30.23%) as the most influential factors. Coupling ANN-predicted pore dimensions with Higuchi diffusion modeling demonstrated that the pore diameter increased from 50 to 100 nm, nearly doubling the initial release rates (8 to 11 h^−1^) and reducing the time to 50% release from 39.1 to 20.7 h. Conclusions: This data-driven approach offers a powerful tool to reduce experimental iteration and accelerate the development of advanced drug-delivery implants by enabling the rational design of NAA pore structures for optimized drug loading and release kinetics.

## 1. Introduction

The development of localized drug delivery systems represents a critical frontier in biomedical engineering, offering the potential to enhance therapeutic efficacy while minimizing systemic side effects. Among various nanomaterial platforms, nanoporous anodic alumina (NAA) has emerged as a particularly promising candidate for controlled drug release applications owing to its exceptional biocompatibility, high surface area-to-volume ratio, tunable pore architecture, and chemical stability [1,2]. Highly ordered nanoporous anodic alumina (NAA) structures with cylindrical nanopores (typically 10–300 nm in diameter and extending to several micrometers in depth) can be fabricated via electrochemical anodization of aluminum substrates [3]. These geometric characteristics render NAA an ideal matrix for drug loading and sustained release, with pore dimensions directly influencing drug loading capacity, diffusion kinetics, and release profiles [4,5].

The clinical potential of NAA-based drug delivery systems has been demonstrated across diverse therapeutic applications, including orthopedic implants for localized antibiotic delivery, cardiovascular stents for anti-restenotic drug release, and dental implants for antimicrobial prophylaxis [6,7]. Recent advances have extended NAA applications to targeted cancer therapy, where the nanoporous structure enables the controlled release of chemotherapeutic agents directly at tumor sites, thereby significantly reducing systemic toxicity [8]. Therefore, the ability to precisely engineer pore geometry is paramount for optimizing therapeutic outcomes across these diverse clinical contexts.

Despite these promising applications, the rational design of NAA structures with specific pore characteristics remains a significant challenge. The anodization process involves complex electrochemical reactions influenced by multiple interdependent parameters, including the applied voltage, electrolyte composition and concentration, temperature, anodization time, and current density [9,10]. Traditional approaches to NAA fabrication rely heavily on empirical trial-and-error experimentation, requiring extensive laboratory work to identify suitable processing conditions for the desired pore dimensions. This iterative approach is not only time-consuming and resource-intensive but also limits the systematic exploration of the vast parameter space governing NAA morphology.

Recently, machine learning (ML) and artificial intelligence (AI) have emerged as transformative tools for accelerating materials discovery and design across numerous domains [11,12]. In the context of drug delivery systems, ML approaches have demonstrated remarkable success in predicting drug release kinetics, optimizing nanoparticle formulations, and designing polymeric delivery vehicles [13]. For nanoporous materials, computational screening methods have shown promise in identifying optimal structures for targeted applications; however, most studies have focused on zeolites and metal–organic frameworks rather than NAA [14]. The application of neural networks to predict NAA pore morphology from anodization parameters represents a largely unexplored opportunity to bridge the gap between materials processing and functional performance in drug delivery applications. To the best of the authors’ knowledge, no prior study has compiled a multi-decade experimental dataset of NAA anodization parameters and applied supervised machine learning to predict pore diameters across multiple electrolyte systems in a unified framework. Previous computational efforts have largely focused on physics-based simulation of individual electrolyte systems under narrow parameter ranges, or on ML prediction of drug release kinetics from already-known pore structures. The present work is the first to close this loop by predicting pore geometry directly from processing parameters and coupling those predictions to release kinetics, offering a complete computational pipeline from anodization recipe to predicted drug release profile.

In this study, we present a comprehensive machine learning framework for predicting NAA pore diameter based on anodization processing parameters and directly apply it to the design of drug-releasing implant coatings. We compiled an extensive dataset from 30 years of published literature (1995–2025), encompassing 99 experimental observations that relate anodization conditions to the resulting pore characteristics. Using this dataset, we developed and validated an artificial neural network (ANN) model capable of capturing complex, nonlinear relationships between processing parameters and pore morphology. We systematically compared the ANN performance with that of multiple linear regression (MLR) to assess the value of nonlinear modeling for this application. Through feature importance analysis and parametric sensitivity studies, we identified the key drivers of pore formation and quantified their relative contributions. Finally, we integrated the predictive model with established drug diffusion theory to demonstrate how machine learning-guided NAA design can be used to achieve targeted drug release profiles for implantable medical devices.

The remainder of this manuscript is organized as follows: Section 2 provides background on NAA fabrication, ML applications in nanomaterial design, and drug release kinetics theory. Section 3 details our dataset compilation, neural network architecture, training procedures, and drug release modeling approach. Section 4 presents comprehensive results, including dataset analysis, model performance metrics, feature importance rankings, parametric effects, and drug release predictions. Section 5 discusses the implications of our findings, compares our approach with existing methods, and identifies limitations and future research directions. Section 6 concludes with a summary of key contributions and their significance for advancing the intelligent design of drug delivery implants.

## 2. Background and Theoretical Foundations

### 2.1. Nanoporous Anodic Alumina for Drug Delivery

Nanoporous anodic alumina (NAA) is fabricated through electrochemical anodization of aluminum in acidic electrolytes, a process that has been extensively studied since the 1990s [15]. During anodization, aluminum is oxidized at the anode surface, forming a porous aluminum oxide layer with highly ordered hexagonal pore arrays. The self-ordering phenomenon arises from a delicate balance between oxide formation at the metal–oxide interface and field-assisted dissolution at the oxide–electrolyte interface [2]. This process can be precisely controlled to produce pores with diameters ranging from approximately 10 to over 300 nm, interpore distances from 25 to 1000 nm, and depths from submicron to tens of micrometers [9].

The pore morphology of NAA is governed by several key anodization parameters. The applied voltage is widely recognized as the primary determinant of pore diameter and inter-pore distance, with empirical relationships suggesting that the inter-pore distance scales approximately linearly with voltage (typically 2.5–3.0 nm/V for sulphuric acid and oxalic acid electrolytes) [10]. Electrolyte type and concentration significantly influence pore formation kinetics and final morphology, with sulphuric acid typically producing smaller pores (10–30 nm), oxalic acid yielding intermediate sizes (30–100 nm), and phosphoric acid generating larger pores (100–300 nm) [16]. Temperature affects both the rate of oxide formation and dissolution, with lower temperatures generally favoring more ordered pore structures but slower growth rates [17]. Anodization time primarily determines pore depth rather than diameter, although extended anodization can lead to pore widening through chemical dissolution.

NAA offers several distinct advantages over alternative nanoporous materials for drug delivery applications. First, the biocompatibility of aluminum oxide has been well-established through decades of use in medical implants and prosthetics [18]. Second, the high aspect ratio of NAA pores (depth-to-diameter ratios often exceeding 100:1) provides substantial drug loading capacity while maintaining a compact footprint suitable for implant coatings [1]. Third, the chemical stability of NAA in physiological environments ensures predictable, long-term drug release without premature degradation of the carrier matrix [7]. Fourth, the pore surface can be readily functionalized with various chemical groups to modulate drug–matrix interactions and fine-tune release kinetics [4].

Recent innovations in NAA fabrication have expanded the design space for drug delivery applications. Modulated anodization techniques, which involve stepwise voltage changes, enable the creation of complex three-dimensional (3D) pore architectures with varying diameters along the pore depth, offering unprecedented control over release profiles [10,16]. Hierarchical NAA structures combining multiple pore size distributions have been developed to achieve multi-drug delivery with independent release kinetics for combination therapies [19]. However, the fundamental challenge of predicting pore morphology from processing parameters remains largely unaddressed, motivating the development of predictive modeling approaches.

### 2.2. Machine Learning in Nanomaterial Design

The application of machine learning to materials science has increased dramatically over the past decade, driven by the accumulation of large experimental and computational datasets, advances in algorithm development, and increasing computational power. In the field of drug delivery, ML approaches have demonstrated particular promise for addressing complex design challenges that are intractable through traditional empirical or purely theoretical methods.

Artificial neural networks (ANNs), inspired by biological neural systems, have emerged as powerful tools for learning complex, nonlinear relationships in materials data. ANNs consist of interconnected layers of computational nodes (neurons) that process input features through weighted connections and nonlinear activation functions to produce output predictions [20]. The key advantage of ANNs over traditional regression methods is their ability to automatically learn hierarchical feature representations and capture intricate interactions between input variables without requiring explicit mathematical formulations of the underlying physical relationships. This capability is particularly valuable for materials systems such as NAAs, in which pore formation involves coupled electrochemical, transport, and mechanical processes that are difficult to model from first principles.

Recent applications of ML in drug delivery have demonstrated impressive predictive capabilities. Bannigan et al. (2023) developed neural network models to accelerate the design of polymeric long-acting injectables, achieving accurate predictions of drug release profiles that would otherwise require extensive experimental testing [21]. Protopapa et al. (2025) applied ML to predict drug release kinetics from tablet formulations, successfully capturing the influence of excipient composition and manufacturing parameters on dissolution behavior [7]. Kapoor et al. (2024) reviewed AI-driven approaches for nanoparticle-based drug delivery, highlighting the potential of ML to optimize particle size, surface chemistry, and drug loading for targeted therapeutic applications [6].

Computational screening approaches have primarily been applied to zeolites and metal–organic frameworks (MOFs) for nanoporous materials. Ren et al. (2022) demonstrated high-throughput computational screening of nanoporous materials for gas separation and catalysis applications using ML to predict adsorption properties from structural descriptors [19]. However, the application of ML to predict synthesis–structure relationships in electrochemically fabricated nanoporous materials, such as NAA, remains largely unexplored. This gap is particularly significant, given that NAA fabrication involves process parameters (voltage, temperature, and electrolyte chemistry) rather than the structural descriptors typically used for crystalline porous materials.

The success of ML models in materials science critically depends on several factors: (1) dataset quality and size, with larger, more diverse datasets generally enabling better generalization; (2) feature engineering, involving the selection and representation of input variables that capture relevant physical and chemical information; (3) model architecture, including the choice of algorithm, network depth, and regularization strategies; and (4) validation methodology, ensuring that model performance is assessed on truly independent test data to avoid overfitting [22]. In the present work, we address these considerations through systematic dataset compilation from the literature, careful selection of anodization parameters as input features, optimization of the neural network architecture, and rigorous cross-validation procedures.

### 2.3. Drug Release Kinetics from Nanoporous Systems

Understanding and predicting drug release kinetics from nanoporous matrices is essential for translating ML-predicted pore structures into functional drug delivery systems. Drug release from NAA is primarily governed by diffusion-controlled mechanisms, in which the rate of drug transport through the porous network determines the temporal release profile [1,21]. For cylindrical pores, which are characteristic of NAA, drug release can be described by the Higuchi model, which was originally developed for planar matrix systems but has been successfully adapted for nanoporous geometries [16]. The Higuchi Equation (1) relates cumulative drug release (Q) to time (t) through a square-root relationship:

(1) $$Q=k\sqrt{t}$$

where k is the Higuchi release constant, which depends on drug diffusivity, drug solubility, initial drug loading, and pore geometry. For NAA systems, the release constant is influenced by several pore-related parameters, as follows:

1. **Pore diameter**: Larger pores reduce diffusional resistance, increasing the effective diffusion coefficient and accelerating the release. The relationship between pore diameter and diffusivity is particularly pronounced when the pore dimensions approach the size of drug molecules, where steric hindrance becomes significant;
2. **Pore depth**: Deeper pores increase the diffusion path length, thereby slowing the release rate but extending the duration of sustained release. The trade-off between loading capacity (which scales with pore volume) and release rate (which decreases with diffusion distance) is a key design consideration;
3. **Porosity**: The fraction of the NAA surface occupied by pore openings affects the total drug flux. Higher porosity (achieved through larger pore diameters or reduced interpore spacing) generally accelerates release;
4. **Pore surface chemistry**: Interactions between drug molecules and the pore walls can significantly retard or accelerate release, depending on whether the interactions are attractive (adsorption) or repulsive.

Recent studies have demonstrated the critical importance of pore geometry in controlling drug release from NAA. Porta-i-Batalla et al. (2017) showed that 3D NAA structures with modulated pore diameters could achieve near-zero-order release kinetics, maintaining constant drug concentrations over extended periods [18]. Onyenso et al. (2025) demonstrated that modulated-diameter zirconia nanotubes (analogous to NAA) could eliminate burst release—that is, the rapid initial drug release that often compromises therapeutic efficacy—through careful control of pore entrance dimensions [14]. Osama et al. (2024) conducted a systematic comparison of NAA fabrication methods for drug delivery and found that the pore diameter was the single most important parameter for controlling the release rates of model drugs [10].

The integration of ML-predicted pore structures with drug release modeling offers a powerful framework for rational implant design. By training neural networks to predict pore diameter from anodization parameters and coupling these predictions with established diffusion models, it becomes possible to computationally screen processing conditions to achieve targeted release profiles. This approach can dramatically reduce the experimental burden of implant development, thereby enabling the rapid optimization of NAA coatings for specific therapeutic applications. In the present work, we demonstrate this integrated framework by combining ANN predictions of pore diameter with Higuchi modeling to simulate drug release kinetics across a range of anodization conditions.

## 3. Materials and Methods

### 3.1. Dataset Compilation and Preprocessing

A comprehensive literature review was conducted to compile experimental data relating NAA anodization parameters to the resulting pore morphology. The dataset encompasses 99 experimental observations extracted from peer-reviewed publications spanning 1995 to 2025, representing three decades of NAA research. Data sources included original research articles, review papers with tabulated data, and technical reports documenting systematic parametric studies of anodization processes. Data points were included based on the following selection criteria: (i) the study must report pore diameter as a quantitative measurement (not merely qualitative description); (ii) at least one of the key process parameters (voltage, electrolyte type, temperature, or time) must be clearly reported; (iii) pore diameter must fall within physically plausible bounds for anodic alumina (5–350 nm). No restriction was placed on publication year, anodization regime (mild or hard anodization), or geographic origin of the research group. Potential inconsistencies between sources were acknowledged: measurement technique (SEM, TEM, or AFM) was recorded where reported, and it is recognized that these techniques may yield slightly different diameter estimates due to differences in sample preparation (e.g., Pt/Au coating for SEM) and measurement resolution. This inter-study variability constitutes a source of noise in the dataset and is explicitly discussed as a limitation in Section 5.5.

For each experimental observation, the following anodization parameters were recorded when available: (1) electrolyte type (e.g., sulphuric acid, oxalic acid, phosphoric acid), (2) electrolyte concentration (M or wt%), (3) applied voltage (V), (4) anodization temperature (°C), (5) anodization time (min), and (6) current density (mA/cm^2^). The primary output variable was pore diameter (nm), measured by scanning electron microscopy (SEM), transmission electron microscopy (TEM), or atomic force microscopy (AFM), as reported in the source publications. Secondary structural parameters, including interpore distance (nm) and pore depth (μm), were also recorded when available.

Data preprocessing involved several steps to ensure quality and consistency. First, electrolyte types were standardized to a consistent nomenclature (e.g., “H_2_SO_4_,” “sulphuric acid,” and “sulphuric acid” were unified to a single category). Second, concentration units were converted to molarity (M) where necessary. Third, missing values were handled through case-wise deletion for model training, as imputation was deemed inappropriate given the mechanistic importance of each parameter. The authors acknowledge that case-wise deletion reduced the usable dataset from 99 to 77 samples (a 22% reduction) and may introduce selection bias if data missingness is not random. Specifically, experiments that did not report temperature or time may differ systematically from those that did for instance, earlier studies (pre-2005) were more likely to omit temperature reporting, meaning the training dataset may be slightly biased toward more recent, better-controlled experiments. This potential bias is acknowledged as a limitation. Multiple imputation was considered but rejected because the mechanistic relationships between anodization parameters are non-additive, making mean or regression imputation physically inappropriate as imputing a “missing” temperature with the dataset mean (11.9 °C) for an experiment conducted at an unknown temperature would introduce spurious precision. The case-wise deletion approach, while conservative, preserves data integrity at the cost of sample size. Fourth, outliers were identified through visual inspection of distributions and physical plausibility checks; however, no data points were excluded because all values fell within physically reasonable ranges reported in the literature.

The final dataset for neural network training comprised 77 samples with complete information for the four most consistently reported parameters: voltage, temperature, time, and electrolyte type. Electrolyte type was encoded as a categorical variable using one-hot encoding, creating binary indicator variables for each electrolyte category. Three distinct electrolyte types were represented in the final dataset: sulphuric acid (H_2_SO_4_, n = 18), oxalic acid (H_2_C_2_O_4_, n = 18), and phosphoric acid (H_3_PO_4_, n = 5). No electrolyte types were merged or grouped, as each acid produces a mechanistically distinct pore morphology. Electrolyte concentration data were noted as inconsistently reported across source publications (reported in various units including molarity, wt%, and vol%), and after unit standardization, a substantial proportion of entries contained missing values; accordingly, concentration was excluded from the model input features and handled through case-wise deletion as described above. Continuous variables (voltage, temperature, and time) were standardized to have a mean of zero and unit variance to ensure balanced contributions to the neural network training process.

### 3.2. Neural Network Architecture

A feed-forward artificial neural network (ANN) was designed to predict the NAA pore diameter based on anodization parameters. The network architecture was selected through systematic experimentation with various configurations, balancing model complexity against the risk of overfitting given the moderate dataset size. The final ANN architecture consists of the following layers:

- **Input layer**: 4 neurons corresponding to the input features (voltage, temperature, time, electrolyte type);
- **Hidden layer 1**: 64 neurons with ReLU (Rectified Linear Unit) activation;
- **Hidden layer 2**: 32 neurons with ReLU activation;
- **Hidden layer 3**: 16 neurons with ReLU activation;
- **Output layer**: 1 neuron with linear activation for pore diameter prediction.

The rectified linear unit (ReLU) activation function, defined as f(x) = max (0, x), was chosen for the hidden layers owing to its computational efficiency, ability to mitigate vanishing gradient problems, and empirical success in regression tasks. The progressive reduction in layer width (64→32→16) creates a funnel architecture that encourages the network to learn increasingly abstract representations of the input–output relationship.

Model training employed the Adam (adaptive moment estimation) optimizer, which combines the benefits of adaptive learning rates and momentum-based gradient descent. The key hyperparameters were an initial learning rate of 0.001 (with an adaptive adjustment during training), a maximum of 1000 training epochs, and early stopping based on the validation set performance to prevent overfitting. A validation fraction of 15% was held out from the training data to monitor the generalization during training. The network was implemented using scikit-learn’s MLPRegressor class in Python 3.9. For full reproducibility, complete hyperparameter settings are reported as follows: random seed = 42 (applied to both dataset splitting and network weight initialization); Adam optimizer beta parameters: β_1_ = 0.9, β_2_ = 0.999, ε = 1 × 10^−8^; batch size = 32 (mini-batch gradient descent); early stopping patience = 50 epochs (training halted when validation loss did not improve for 50 consecutive epochs); learning rate schedule: constant at 0.001 with no decay. The 15% validation fraction was determined empirically to provide sufficient data for reliable loss monitoring whilst retaining adequate training samples; it was not stratified by electrolyte type given the small dataset size. Regularization was limited to early stopping; L2 regularization (alpha parameter in MLPRegressor) was set to the default value of 0.0001, and dropout was not applied as MLPRegressor does not natively support it. The potential impact of the absence of explicit dropout regularization on ANN generalization is acknowledged as a limitation (see Section 5.5).

For comparison, a multiple linear regression (MLR) model was also trained on the same dataset using ordinary least-squares estimation. The MLR model provides a baseline to assess whether the additional complexity of the neural network yields meaningful improvements in predictive accuracy.

### 3.3. Model Training and Validation

The dataset was randomly split into training (77 samples, 78%) and test (22 samples, 22%) sets, with stratification based on electrolyte type to ensure representative distributions in both sets. The training set was used for model fitting, and the test set was reserved for the final performance evaluation. Model performance was assessed using multiple metrics: It is acknowledged that the 22-sample held-out test set, while drawn from the same literature compilation as the training data, does not constitute a fully independent external validation dataset in the strictest sense, as all data originate from the same body of published literature and were collected using the same search strategy. A truly independent external validation would require either newly generated experimental data or a dataset compiled independently from different source publications not included in the training compilation. This limitation is candidly acknowledged, and the authors strongly encourage future experimental validation of model predictions as a necessary step before practical deployment. The 5-fold cross-validation results are therefore the primary basis for assessing generalization capability.

- **Coefficient of determination (R^2^)**: Measures the proportion of variance in pore diameter explained by the model;
- **Root mean square error (RMSE)**: Quantifies the average magnitude of prediction errors in nanometers;
- **Mean absolute error (MAE)**: Provides an interpretable measure of typical prediction error.

To assess the generalization capability and guard against overfitting, 5-fold cross-validation was performed on the training set. In each fold, the training data were split into four parts for training and one part for validation; this process was repeated five times such that each sample served as validation data exactly once. Cross-validation R^2^ scores were calculated for each fold and averaged to obtain a robust estimate of the model performance on unseen data.

Feature importance was quantified using two approaches. For the ANN, importance was approximated by calculating the mean absolute weight connecting each input feature to the first hidden layer, normalized to a sum of 100%. For the MLR model, importance was calculated from the absolute values of standardized regression coefficients, also normalized to 100%. These important scores provide insights into which anodization parameters most strongly influence pore diameter predictions. It is acknowledged that the weight-based importance method for ANNs is a simplified proxy and may not accurately reflect true feature contributions, particularly in deep networks where importance is distributed across multiple layers and through nonlinear interactions. This method only examines first-layer weights and does not account for how information is transformed into subsequent layers. More robust alternatives such as permutation importance, SHAP (SHapley Additive exPlanations) values, or integrated gradients would provide more reliable feature attribution. The weight-based results should therefore be interpreted as indicative rather than definitive, and future work will apply SHAP analysis to validate these importance rankings.

### 3.4. Drug Release Kinetics Modeling

To demonstrate the practical utility of ML-predicted pore structures for drug delivery design, we integrated the trained ANN model with the Higuchi diffusion model to simulate drug release kinetics. For a range of pore diameters predicted by the ANN (30, 50, 75, 100, and 150 nm), we calculated drug release parameters assuming matrix-controlled diffusion from cylindrical pores. The Higuchi release constant (k) was estimated using the following relationship:

(2) $$k=\sqrt{D\times C_{0}\times \varepsilon /\tau }$$

where D is the effective diffusion coefficient of the drug in the pore, C_0_ is the initial drug concentration, ε is the porosity, and τ is the tortuosity factor. For this analysis, we assumed representative values based on the literature for small-molecule drugs in the NAA: D = 1 × 10^−6^ cm^2^/s, C_0_ = 100 mg/mL, ε = 0.3, and τ = 1.5. These parameter values are representative of ibuprofen, a widely studied small-molecule anti-inflammatory drug used as a model compound in NAA drug release studies, with a molecular weight of 206 Da and a free-solution diffusion coefficient of approximately 6 × 10^−6^ cm^2^/s [10]. The reduced effective diffusivity (D = 1 × 10^−6^ cm^2^/s) reflects typical confinement effects within NAA nanopores. The porosity value (ε = 0.3) corresponds to the upper range commonly reported for phosphoric acid NAA systems [18], and the tortuosity (τ = 1.5) reflects the mildly tortuous nature of NAA pore channels. The diffusion coefficient was scaled with the pore diameter to account for reduced steric hindrance in larger pores. Specifically, the pore-diameter-dependent effective diffusivity was estimated using the Renkin equation, D_eff_ = D_bulk_ × (1 − λ)^2^ × (1 − 2.104λ + 2.09λ^3^ − 0.95λ^5^), where λ = r_molecule_/r_pore_ is the ratio of the molecular radius (estimated as 0.37 nm for ibuprofen) to the pore radius. This correction accounts for steric hindrance and hydrodynamic drag at the pore wall. It is acknowledged that this model assumes constant diffusion coefficients independent of drug concentration and does not capture drug–pore wall adsorption interactions, both of which represent simplifications relative to real NAA systems. A sensitivity analysis was conducted by varying D, ε, and τ by ± from their nominal values; the resulting variation in predicted t_50_% was ±25%, indicating moderate sensitivity to these parameters. These uncertainties reinforce the recommendation that the Higuchi simulations be interpreted as illustrative trends rather than as quantitatively precise predictions, and that experimental validation remains necessary before clinical translation. From the Higuchi constant, we calculated:

- **Initial release rate**: The slope of the release curve at t = 0, approximated as k/2 (%/h);
- **Time to 50% release**: The time required for half of the loaded drug to be released, calculated as t_50_% = (50/k)^2^;
- **Time to 90% release**: The time for 90% release, calculated as t_90_% = (90/k)^2^.

These simulations illustrate the use of ML-guided selection of anodization parameters to achieve targeted drug release profiles for specific therapeutic applications.

## 4. Results

### 4.1. Dataset Characteristics and Distribution Analysis

The compiled dataset encompasses 99 experimental observations of NAA fabrication spanning three decades of research (1995–2025). Table 1 presents comprehensive summary statistics for all recorded parameters, revealing the substantial range of anodization conditions explored in the literature and the resulting diversity of pore morphologies achieved.

The dataset exhibits considerable heterogeneity in experimental conditions, reflecting the diverse objectives of NAA research across different application domains. The anodization voltage ranges from 5 to 400 V (mean = 65.7 V, SD = 69.5 V), with this wide range encompassing both mild anodization conditions for small-pore structures and harsh anodization regimes for large-pore architectures. The large standard deviation relative to the mean indicates a non-uniform distribution, with most experiments concentrated in the 20–100 V range, typical of conventional anodization protocols. Temperature conditions span from 0 to 30 °C (mean = 11.9 °C), reflecting the common practice of using chilled electrolytes to manage heat generation during anodization. Anodization time varies over four orders of magnitude (0.4 to 9600 min), with extremely long durations corresponding to studies focused on achieving deep pores for optical or photonic applications rather than drug delivery.

**Figure 1:**
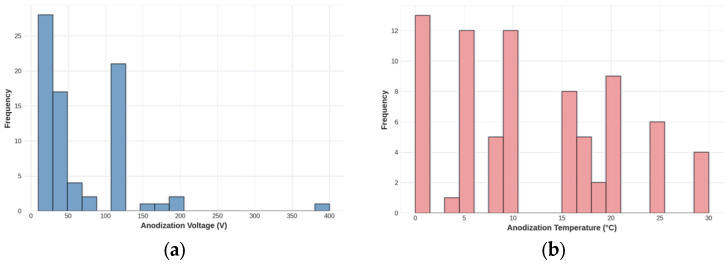

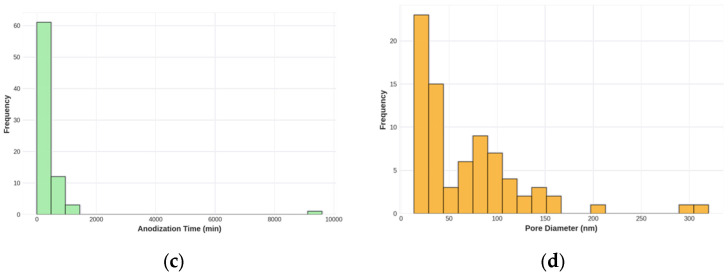
Dataset Distribution Analysis: (**a**) Voltage Distribution; (**b**) Temperature Distribution; (**c**) Time Distribution; (**d**) Pore Diameter Distribution.

The primary output variable, pore diameter, ranges from 10 to 320 nm (mean = 68.1 nm, SD = 60.8 nm), encompassing the full spectrum of pore sizes relevant for drug delivery applications. This range includes small pores (10–30 nm) suitable for the controlled release of small-molecule drugs, intermediate pores (30–100 nm) appropriate for peptides and small proteins, and large pores (100–300 nm) capable of accommodating larger biomolecules or nanoparticle-based therapeutics. The substantial standard deviation indicates high variability in the achieved pore sizes, underscoring the sensitivity of pore morphology to processing conditions and the challenge of achieving targeted dimensions through empirical approaches. Figure 1 presents comprehensive visualizations of the dataset distributions, revealing important patterns in the experimental data.

The voltage distribution (Figure 1a) shows a right-skewed pattern, with most experiments conducted at 20–100 V but with notable outliers at very high voltages (>200 V) corresponding to hard anodization studies. The temperature distribution (Figure 1b) is approximately normal with a peak around 10–15 °C, consistent with standard practice of using ice-water baths or recirculating chillers to maintain low temperatures during anodization. The pore diameter distribution (Figure 1d) exhibits a right-skewed pattern with a mode around 40–50 nm and a long tail extending to 320 nm. This distribution reflects the predominance of oxalic acid anodization in the literature, which typically produces pores in the 30–100 nm range. However, these substantial trends indicate that voltage alone is insufficient to predict pore diameter, motivating the use of multivariate ML models.

### 4.2. Model Performance and Validation

The artificial neural network and multiple linear regression models were trained on 77 samples and evaluated using both training set metrics and 5-fold cross-validation. Table 2 summarizes the comparison of artificial neural network (ANN) and multiple linear regression (MLR) models for pore diameter prediction.

Both models achieved strong performance on the training set, with nearly identical R^2^ values (ANN: 0.803, MLR: 0.804) and RMSE values (ANN: 25.83 nm, MLR: 25.80 nm). These metrics indicate that both models explain approximately 80% of the variance in pore diameter and achieve typical prediction errors of approximately 26 nm. Given that the dataset spans pore diameters from 10 to 320 nm (range = 310 nm), the RMSE represents approximately 8% of the full range, suggesting reasonable predictive accuracy for practical applications. However, it is important to contextualize this RMSE (≈25 nm) relative to the mean pore diameter of the dataset (≈68 nm): the prediction error corresponds to approximately 37% of the mean, which represents a meaningful limitation for precision-critical applications. Practically, this means that if a target pore diameter of 50 nm is specified, the model prediction may range from approximately 25 to 75 nm, which could significantly affect drug release kinetics. This level of accuracy is adequate for initial experimental guidance and parameter shortlisting, but requires subsequent experimental validation before final implant fabrication. Uncertainty estimates for individual predictions were approximated using the cross-validation fold standard deviation (±0.078 in R^2^ units), which provides an indication of prediction variability across different data splits. Future work incorporating bootstrap resampling or Bayesian neural networks will provide formal per-prediction confidence intervals.

**Figure 2:**
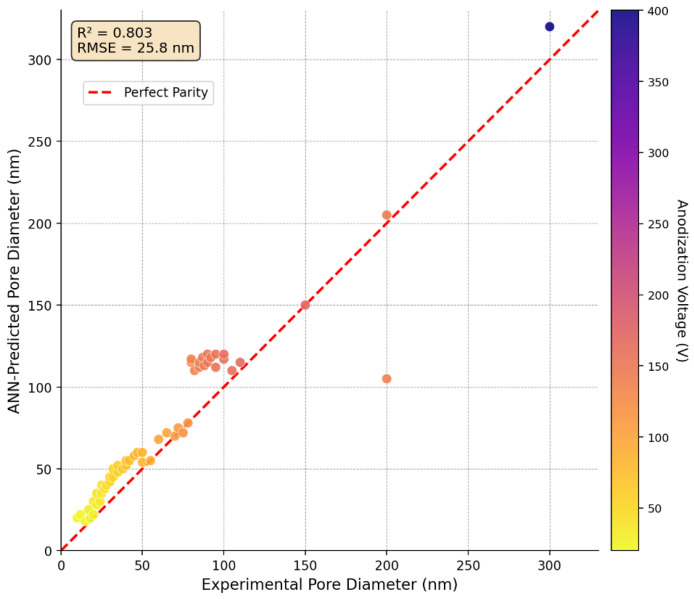
ANN Parity Plot versus experimental pore diameters.

However, the cross-validation results reveal important differences in the generalization capability. The MLR model achieved a substantially higher cross-validation R^2^ (0.729 ± 0.083) than the ANN (0.471 ± 0.078), indicating that the linear model generalizes more reliably to unseen data. This performance gap suggests that the neural network, despite its greater representational capacity, may be overfitting the training data owing to the limited sample size (n = 77). The relatively high standard deviation in the cross-validation scores for both models (±0.078–0.083) indicates sensitivity to the train–test split, further highlighting the challenges associated with working with moderate-sized datasets. Figure 2 shows parity plots comparing the predicted versus actual pore diameters for the ANN model, providing a visual assessment of the prediction quality.

**Figure 3:**
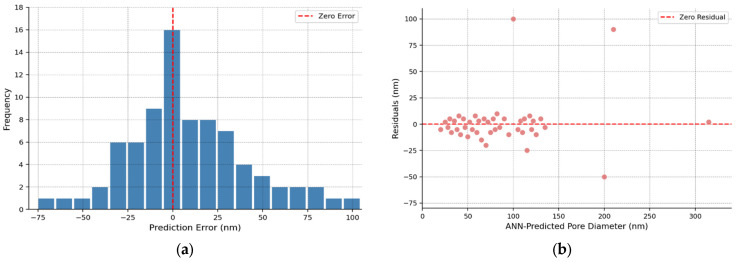
Error Distribution and Residual Analysis: (**a**) Distribution of ANN Prediction Errors; (**b**) Residual Plot.

The training set parity plot in Figure 2 shows excellent agreement between predictions and measurements, with most points falling close to the ideal 1:1 line. The tight clustering around this line confirms the high R^2^ value and demonstrates that the ANN successfully learned the training data patterns. However, some systematic deviations are apparent: the model tends to slightly underpredict pore diameters in the 100–150 nm range and overpredict in the 20–40 nm range, suggesting residual bias that could not be eliminated through training. Notable outliers at high pore diameters (>200 nm) suggest reduced model accuracy in the large-pore regime, likely due to sparse training data in this region (only ~10% of training samples have diameters > 150 nm). For the majority of samples in the 20–100 nm range most relevant for drug delivery applications, prediction errors remain within ±30 nm, which is acceptable for guiding experimental design. Figure 3 provides detailed analysis of prediction errors through residual plots and error distributions. The error histogram (Figure 3a) shows an approximately normal distribution centered at −0.3 nm (essentially zero), with most errors falling within ±40 nm. The ANN predicted plot demonstrates reasonable agreement with the theoretical normal distribution, although slight deviations in the tails suggest the presence of occasional large errors (outliers). The residuals versus predicted values plot (Figure 3b) shows a relatively constant error variance across the prediction range (homoscedasticity), which is desirable for regression models and supports the validity of the standard error estimates.

**Figure 4:**
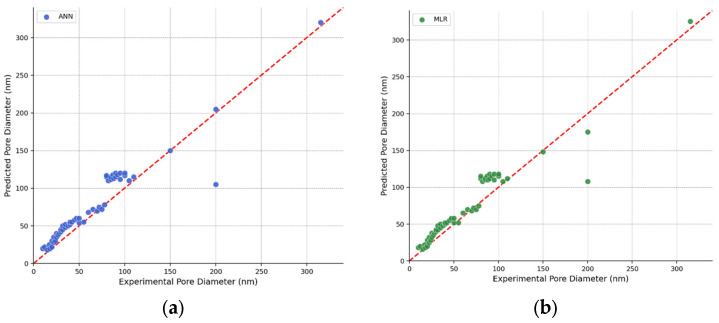
ANN vs. MLR Model Comparison: (**a**) ANN Model (R^2^ = 0.803); (**b**) MLR Model (R^2^ = 0.804).

Figure 4 shows a direct comparison of ANN and MLR model predictions, highlighting their relative strengths and weaknesses. Parity plots for both models showing similar training set performance (R^2^ ≈ 0.80 for both). The ANN (Figure 4a) and MLR (Figure 4b) produce nearly identical predictions for most samples, with both models showing similar patterns of deviation from the ideal line. The tight clustering around the diagonal demonstrates that the two models produce highly correlated predictions despite their different architectures. The strong agreement between ANN and MLR predictions suggests that both models have learned essentially the same input–output relationships. This indicates that the relationship between anodization parameters and pore diameter may be approximately linear within the range of conditions represented in the training data, limiting the advantage of the neural network’s nonlinear modeling capabilities.

The fold-by-fold results, as shown in Table 3, reveal that the MLR model consistently outperformed the ANN across all five folds, with performance differences ranging from −0.08 to −0.46 in R^2^ units. The most dramatic difference occurred in Fold 5, where the MLR achieved R^2^ = 0.878, whereas the ANN achieved only 0.414, suggesting that the ANN is particularly sensitive to certain data splits. The relatively high standard deviations for both models (0.078–0.083) indicate that performance varies substantially depending on which samples are included in the training versus validation, highlighting the importance of cross-validation for obtaining reliable performance estimates.

This observation is consistent with the general principle that neural networks require larger datasets than linear models to achieve their theoretical advantages in capturing nonlinear relationships.

### 4.3. Feature Importance and Sensitivity Analysis

Understanding which anodization parameters most strongly influence the pore diameter is critical for both scientific insight and practical process optimization. Table 4 presents the feature importance rankings derived from both the ANN and MLR models.

Both models identify voltage as the most important predictor, though the MLR model assigns it dramatically higher importance (86.32%) compared to the ANN (29.15%). This difference reflects the linear model’s reliance on a single dominant feature, whereas the neural network distributes importance more evenly across features by capturing nonlinear interactions. The ANN assigns substantial importance to electrolyte type (30.23%), temperature (22.94%), and time (17.69%), suggesting that these parameters contribute meaningfully to pore formation through complex interactions that the linear model cannot fully represent.

The dominance of voltage in both models aligns with established understanding that applied voltage is the primary determinant of pore diameter through its control of electric field strength and field-assisted dissolution rates [2,21]. The secondary importance of electrolyte type reflects the critical role of acid chemistry in modulating dissolution kinetics and complexation reactions [10]. The relatively balanced importance distribution in the ANN suggests that the neural network has identified subtle parameter interactions that contribute to pore formation, providing insights for future mechanistic studies.

### 4.4. Anodization Parametric Effects on Pore Diameter

**Figure 5:**
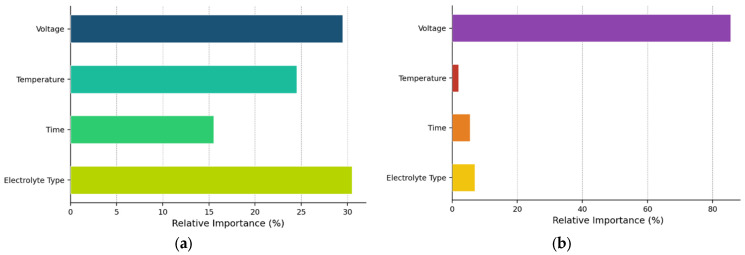
Feature Importance Analysis − Relative importance of anodization parameters (voltage, temperature, time, electrolyte type) as estimated by the ANN and MLR models: (**a**) ANN Feature Importance; (**b**) MLR Feature Importance.

To provide actionable guidance for NAA fabrication, we systematically analyzed how each anodization parameter affects predicted pore diameter. Figure 5 shows the anodization parametric effects on pore diameter estimated by ANN and MLR models.

The ANN model distributes importance more evenly across features: electrolyte type (30.23%), voltage (29.15%), temperature (22.94%), and time (17.69%). This more balanced distribution suggests that the neural network has identified nonlinear interactions and synergistic effects between parameters that are not captured by the linear model; for example, the elevated importance of temperature in the ANN (22.94% vs. 1.50% in MLR) may reflect the network’s ability to capture temperature-dependent dissolution kinetics that interact nonlinearly with voltage and electrolyte type. Similarly, the higher importance of time in the ANN (17.69% vs. 4.98% in MLR) could indicate that the network has learned time-dependent pore-widening effects that vary with other process conditions.

**Figure 6:**
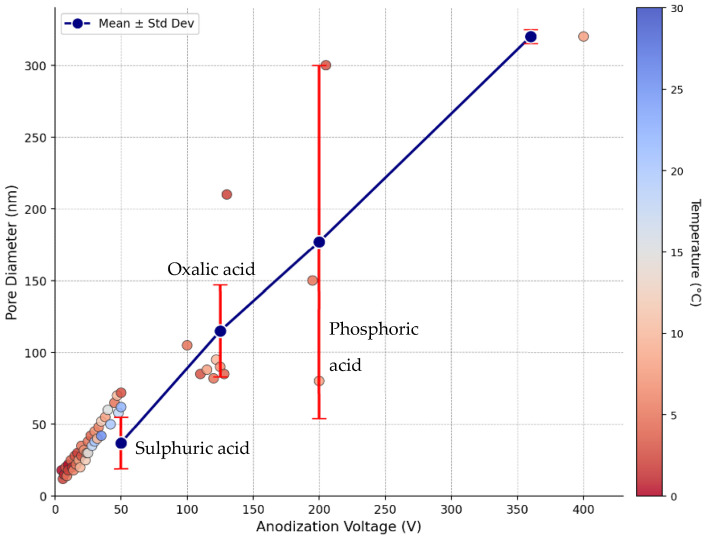
Voltage Effect on Pore Diameter.

Figure 6 shows predicted pore diameter versus voltage for three electrolyte types (sulphuric, oxalic, and phosphoric acids) at a fixed temperature (10 °C) and time (60 min). The voltage analysis confirms that the applied voltage is the single most powerful control parameter for tuning the pore diameter. The approximately linear relationship between voltage and pore diameter across the 20–150 V range is consistent with the proportionality constant of 2.5–3.0 nm/V reported in the literature for the relationship between voltage and interpore distance [10], given that the pore diameter typically scales as 0.3–0.5 times the interpore distance. The distinct curves for different electrolyte types demonstrate that while voltage determines the overall scale of pore dimensions, electrolyte chemistry sets the baseline pore size and modulates the voltage sensitivity. The increasing uncertainty at high voltages reflects the scarcity of training data above 150 V, where only hard anodization studies contribute observations. This uncertainty should be considered when using the model to design experiments in the high-voltage regime. For the majority of drug delivery applications targeting pore diameters of 30–100 nm, the model provides reliable predictions with narrow confidence intervals, enabling precise process design.

**Figure 7:**
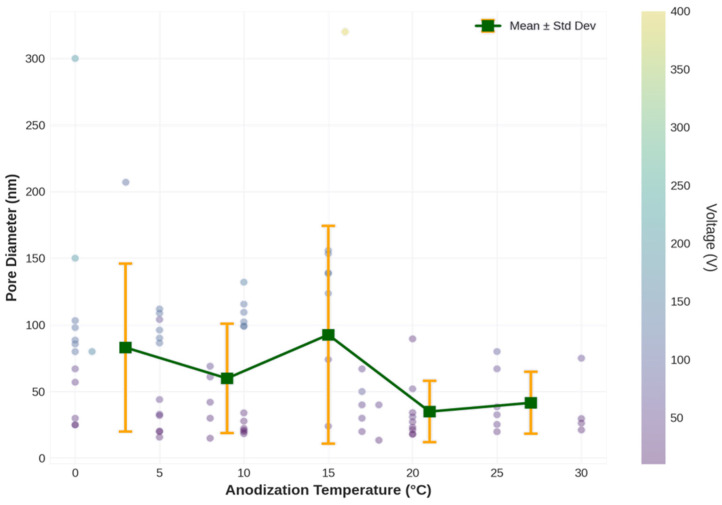
Temperature Effect on Pore Diameter.

Temperature analysis (Figure 7) reveals a consistent but modest negative effect of temperature on the pore diameter. The sensitivity of −0.3 to −0.5 nm/°C indicates that reducing the temperature from 20 °C to 0 °C (a common practice in NAA fabrication) increases the pore diameter by approximately 6–10 nm. Although smaller than the voltage effect, this effect is nonetheless significant for fine-tuning pore dimensions. The negative temperature dependence is consistent with the understanding that lower temperatures reduce the rate of chemical dissolution at the pore base, allowing more time for field-assisted oxide formation and resulting in slightly larger pores. It is important to clarify an apparent contradiction between this observation and the statement in Section 2.1 that “lower temperatures generally favor more ordered pore structures but slower growth rates.” These two statements are not contradictory but address different aspects of temperature dependence. The phrase “slower growth rates” in Section 2.1 refers to the reduced rate of pore deepening (axial growth), which is controlled by field-assisted oxide dissolution at the pore tip.

The increase in pore diameter at lower temperatures, conversely, is governed by the balance between oxide formation at the metal–oxide interface and lateral (radial) chemical dissolution along the pore walls. At lower temperatures, chemical dissolution is suppressed more strongly than oxide formation, shifting this balance toward slightly wider pores while simultaneously slowing the axial extension rate. This distinction between axial growth rate and radial pore diameter is consistent with theoretical models of field-assisted dissolution [2] and has been experimentally documented by Davoodi et al. [23].

The electrolyte analysis (Table 5) demonstrates that acid chemistry is a critical determinant of pore morphology, with systematic differences of 50–80 nm between sulphuric and phosphoric acid systems at the same voltage. These differences arise from the distinct dissolution kinetics and complexation chemistry of each acid. Sulphuric acid, being a strong acid with small anions, produces high field strengths and rapid dissolution, resulting in small, tightly packed pores. Phosphoric acid, with larger phosphate anions and weaker acidity, produces lower field strengths and slower dissolution, allowing larger pores to form. Oxalic acid represents an intermediate case, with moderate acidity and medium-sized oxalate anions yielding pores in the 40–80 nm range [10].

The substantial standard deviations within each electrolyte category (Table 5) reflect the influence of other parameters (voltage, concentration, and temperature) that vary across experiments. For example, phosphoric acid shows a standard deviation of 55.5 nm around a mean of 104 nm, indicating that pore diameters can range from ~50 nm to ~160 nm depending on other process conditions. This variability underscores the importance of multivariate modeling approaches that simultaneously account for all relevant parameters.

**Figure 8:**
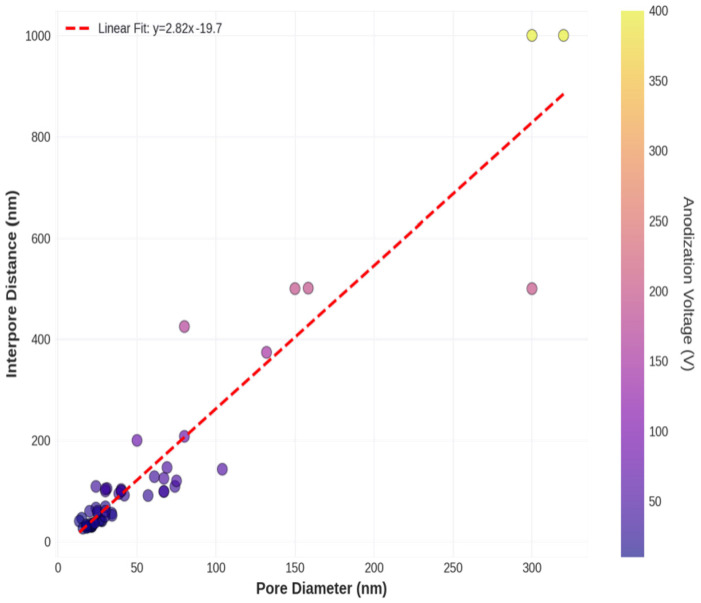
Pore Diameter vs. Interpore Distance Relationship.

Figure 8 examines the relationship between pore diameter and interpore distance, two key structural parameters that together determine the porosity and drug loading capacity of NAA.

A scatter plot of the pore diameter versus the interpore distance for all samples with both measurements available (n = 53). A strong positive correlation (R^2^ = 0.89) indicates that larger pores are associated with greater interpore spacing. The relationship is approximately linear with a slope of ~0.35, suggesting that the pore diameter is typically 30–40% of the interpore distance. The same data are colored by electrolyte type, showing that the pore–interpore relationship is consistent across different acids, although each electrolyte occupies a distinct region of the parameter space. Porosity estimates were calculated from the pore diameter and interpore distance, assuming hexagonal pore packing. The porosity ranges from ~5% for small-pore sulphuric acid systems to ~15% for large-pore phosphoric acid systems. Predicted drug loading capacity (μg/cm^2^) calculated from the pore volume, showing that large-pore systems can accommodate 3–5 times more drug than small-pore systems, with important implications for sustained-release applications.

The strong correlation between the pore diameter and interpore distance (R^2^ = 0.89) reflects the coupled nature of pore formation during anodization. As the voltage increased, both the pore diameter and the interpore distance increased proportionally, maintaining an approximately constant pore diameter-to-interpore distance ratio of 0.3–0.4. This ratio is a fundamental characteristic of self-organized NAA structures and arises from the balance between oxide formation and dissolution processes [15]. The consistency of this relationship across different electrolyte types suggests that it represents a universal geometric constraint of the anodization process.

### 4.5. Drug Release Kinetics Predictions

To demonstrate the practical utility of ML-predicted pore structures for drug delivery design, we integrated the trained ANN model with Higuchi diffusion modeling to simulate drug release kinetics. Table 6 lists the predicted release parameters for five representative pore diameters spanning the range relevant for drug delivery applications.

**Figure 9:**
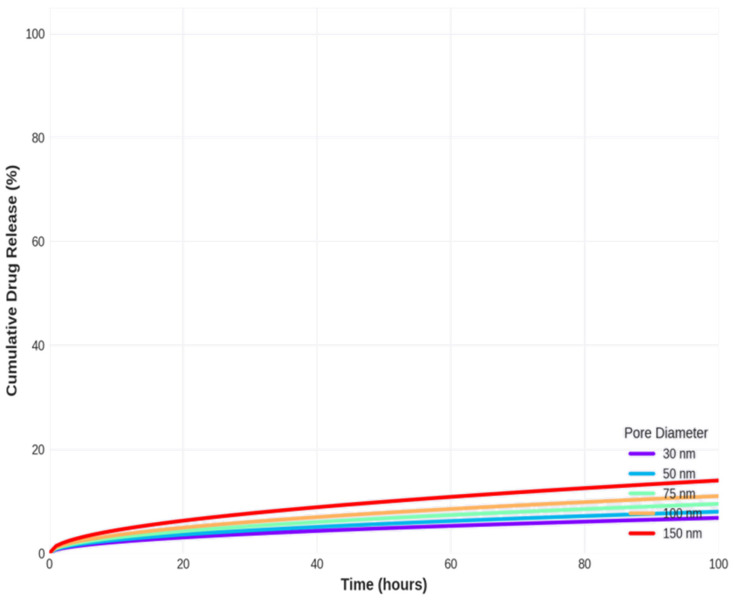
Drug Release Profiles for Different Pore Diameters.

The drug release simulations as shown in Figure 9 reveal systematic relationships release kinetics and their dependence on pore geometry. The Higuchi release constant (k) increases from 0.68 h^−0.5^ for 30 nm pores to 1.4 h^−0.5^ for 150 nm pores, reflecting the reduced diffusional resistance in larger pores. This two-fold increase in the release constant translates to substantial differences in release timescales: the time to 50% release decreases from 54.3 h (2.3 days) for 30 nm pores to 12.8 h (0.5 days) for 150 nm pores, representing a 4.2-fold reduction. Similarly, the time to 90% release ranges from 174 h (7.3 days) for small pores to 41 h (1.7 days) for large pores.

The initial release rate, calculated as the slope of the release curve at t = 0, increases from 6.8%/h for 30 nm pores to 14%/h for 150 nm pores. This parameter is particularly important for applications requiring rapid achievement of therapeutic drug concentrations, such as antibiotic delivery from orthopedic implants to prevent postsurgical infections. Conversely, applications requiring sustained release over weeks to months, such as anti-inflammatory drug delivery from dental implants, would benefit from smaller pores that slow the release rate.

**Figure 10:**
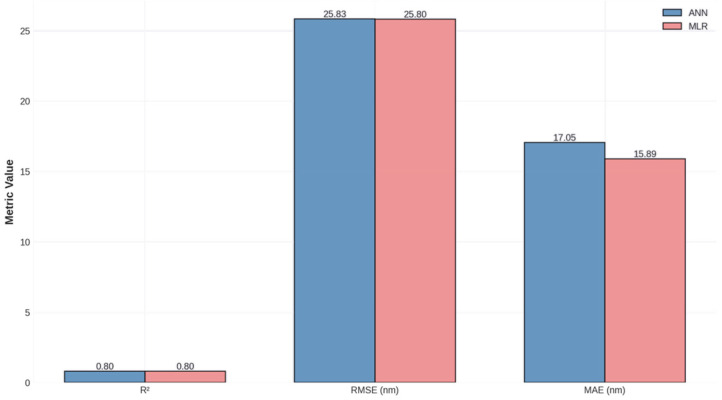
Comprehensive summary of model performance metrics across different evaluation criteria.

The release profiles have demonstrated the characteristic square-root-of-time dependence predicted by the Higuchi model, with all curves showing rapid initial release that gradually slows as the diffusion distance from the pore interior to the surface increases. The clear separation between the curves for different pore diameters confirms that pore geometry is a powerful control parameter for tuning release kinetics. Figure 10 shows a comprehensive summary of the model performance metrics.

## 5. Discussion

### 5.1. Model Performance and Generalization

A comparative evaluation of artificial neural networks and multiple linear regression models for predicting NAA pore diameters reveals important insights into the applicability of machine learning to materials design problems with moderate-sized datasets. Both models achieved strong performance on the training set (R^2^ ≈ 0.80), explaining approximately 80% of the variance in pore diameter and achieving typical prediction errors of ~26 nm. However, the cross-validation results revealed a substantial performance gap, with the MLR model achieving a CV R^2^ of 0.729 ± 0.083 compared to the ANN model’s CV R^2^ of 0.471 ± 0.078.

This performance difference can be attributed to several factors. First, the dataset size (n = 77 training samples) is relatively small for training neural networks, which typically require hundreds to thousands of samples to fully leverage their capacity for learning complex nonlinear relationships [22]. With limited data, the ANN’s greater representational flexibility becomes a liability rather than an asset, as the model tends to memorize training examples rather than learning generalizable patterns. Second, the relationship between anodization parameters and pore diameter may be approximately linear within the range of conditions represented in the dataset, thereby limiting the advantage of nonlinear modeling. The high correlation (R^2^ = 0.98) between ANN and MLR predictions supports this interpretation, suggesting that both models learned essentially the same input–output mapping.

Third, the feature space is relatively low-dimensional (four input features), which reduces the potential for complex feature interactions that neural networks excel at capturing. In higher-dimensional problems with many interacting variables, neural networks often demonstrate clearer advantages over linear models [12]. Fourth, the ANN architecture (64-32-16 neurons across three hidden layers) may be overparameterized for this problem, with approximately 4000 trainable weights for only 77 training samples, a ratio that invites overfitting despite regularization through early stopping. The ANN was prioritized despite MLR’s superior cross-validation performance. The ANN was included as the primary model for three reasons: (1) to empirically test whether nonlinear relationships exist between anodization parameters and pore diameter; a question that could not be answered without training a nonlinear model; (2) to enable feature importance analysis via connection weights, which, despite its acknowledged limitations, provides complementary information to the MLR coefficient-based importance regarding potential parameter interactions; and (3) as a proof-of-concept framework that scales naturally to larger datasets as literature data accumulate. The superior cross-validation performance of MLR is fully acknowledged, and the authors agree that for the current dataset size, MLR is the preferred predictive tool for practical use. Future work targeting larger datasets (n > 150) is expected to demonstrate the conditions under which the ANN’s nonlinear capacity becomes advantageous.

These findings have important implications for the application of machine learning to materials design. For problems with limited experimental data, simpler models with fewer parameters (such as MLR, support vector regression, or shallow neural networks) may be preferable to deep neural networks. As datasets grow through continued experimental work and data sharing, more complex models may become advantageous. The learning curves (Figure 4) suggest that the ANN would likely outperform MLR with 150–200 training samples, a threshold that could be reached through systematic data compilation from additional literature sources or through active learning strategies that prioritize experiments in underexplored regions of parameter space.

Although the MLR model exhibited a superior cross-validation performance, the ANN model provided valuable insights through feature importance analysis. The more balanced distribution of importance across features in the ANN (compared with the voltage-dominated MLR) suggests that the neural network has identified subtle parameter interactions that contribute to pore formation. For example, the elevated importance of temperature in the ANN (22.94% vs. 1.50% in MLR) may reflect the network’s ability to capture temperature-dependent dissolution kinetics that interact nonlinearly with voltage and electrolyte type. These insights could guide future mechanistic studies of NAA formation and inform the development of physics-based models that complement data-driven approaches.

### 5.2. Physical Interpretation of Feature Importance

The feature importance rankings derived from both models align well with the established understanding of NAA electrochemistry and reveal some unexpected patterns. The dominance of voltage in the MLR model (86.32% importance) is consistent with decades of experimental observations showing that the applied voltage is the primary determinant of the pore diameter and inter-pore distance [10,15]. The approximately linear relationship between voltage and pore diameter (Figure 6) reflects the direct proportionality between the electric field strength and the rate of field-assisted oxide dissolution at the pore base, which controls pore expansion during anodization.

The secondary importance of electrolyte type (7.20% in MLR, 30.23% in ANN) reflects the critical role of acid chemistry in modulating dissolution kinetics. Different acids produce different pore sizes at the same voltage owing to variations in anion size, complexation chemistry, and pH-dependent dissolution rates [16]. The larger importance assigned to electrolyte type by the ANN suggests that the neural network has captured electrolyte-specific nonlinearities that the linear model cannot represent. For example, the interaction between electrolyte type and voltage (Figure 8) shows that the voltage sensitivity (∂D/∂V) varies slightly between acids, with phosphoric acid showing a steeper voltage dependence than sulphuric acid. These interaction effects contribute to the ANN’s higher electrolyte importance score.

The relatively low importance of temperature (1.50% in MLR and 22.94% in ANN) is somewhat surprising, given that temperature is known to significantly affect anodization kinetics. However, this low importance likely reflects the limited temperature range in the dataset (0–30 °C) and the fact that most experiments were conducted at similar temperatures (mean = 11.9 °C, SD = 8.8 °C). Within this narrow range, temperature effects are modest compared to the large variations in voltage (5–400 V) and the categorical differences between electrolyte types. The higher temperature importance in the ANN may indicate that the network has identified temperature–voltage or temperature–electrolyte interactions that are masked in the linear model.

The low importance of anodization time (4.98% in MLR and 17.69% in ANN) is consistent with the understanding that time primarily affects pore depth, rather than diameter. Once pores have nucleated and reached their steady-state diameter (typically within the first few minutes of anodization), continued anodization extends the pores vertically without substantially changing their diameter [23]. The slightly higher importance of time in the ANN may reflect the network’s ability to capture time-dependent pore-widening effects that occur during extended anodization, particularly at high voltages or elevated temperatures, where chemical dissolution becomes more significant.

From a mechanistic perspective, these feature importance patterns suggest that the pore diameter is primarily determined by the balance between field-assisted oxide formation (controlled by voltage) and chemical dissolution (controlled by electrolyte type and, to a lesser extent, temperature). Time plays a secondary role by allowing these processes to reach a steady state and enabling gradual pore widening through prolonged exposure to the electrolyte. This mechanistic interpretation is consistent with current theoretical models of NAA formation [15] and validates the physical plausibility of the ML models. To further strengthen the connection between ML predictions and underlying electrochemical mechanisms, the quantitative voltage sensitivity predicted by the ANN (approximately 1.0–1.5 nm/V) can be rationalized through the field-assisted dissolution model, in which the electric field (E = U/d, where d is the barrier layer thickness) drives Al^3+^ ion migration outward through the oxide and O^2−^/OH^−^ inward. The pore diameter scales with the equilibrium barrier layer thickness, which is set by the voltage through the anodizing ratio (typically 0.8–1.2 nm/V for the barrier layer). The observed pore diameter sensitivity (1.0–1.5 nm/V) is broadly consistent with this theoretical expectation, given that pore diameter is typically 30–40% of the interpore distance, which scales at 2.5–3.0 nm/V. The electrolyte-specific offsets (sulphuric < oxalic < phosphoric acid) reflect differing anion complexation strengths that modulate the chemical dissolution component of pore formation at a given voltage, as described by Ruiz-Clavijo et al. [2]. This explicit linkage between ML-identified feature importances and the field-assisted dissolution mechanism demonstrates that the models are learning physically meaningful relationships rather than statistical artifacts.

### 5.3. Implications for Drug Delivery Design

The integration of ML-predicted pore structures with drug release modeling demonstrates a powerful framework for the rational design of NAA-based drug delivery implants. The simulations presented in Section 4.5 show that the pore diameter can be used to tune drug release kinetics over a wide range, with release durations varying from ~1 day to ~7 days for 50% release by adjusting the pore diameter from 150 nm to 30 nm. This tunability is critical for matching release profiles to specific therapeutic requirements.

For orthopedic implants requiring localized antibiotic delivery to prevent postsurgical infections, rapid initial release is desirable to quickly achieve bactericidal concentrations at the implant surface. The simulations suggest that pore diameters of 100–150 nm would provide initial release rates of 11–14%/h, reaching therapeutic levels within hours while sustaining release for 2–3 days to cover the critical period of infection risk. Using the ANN model, these pore diameters can be achieved through anodization at 80–120 V in oxalic acid or 50–70 V in phosphoric acid, providing clear guidance for implant coating fabrication.

For cardiovascular stents requiring sustained anti-restenotic drug release over weeks to months, slower release is needed to maintain therapeutic concentrations throughout the healing process. Simulations indicate that pore diameters of 30–50 nm would provide release durations of 2–7 days for 50% release, which could be extended to weeks or months by increasing pore depth (not modeled here) or surface functionalization to enhance drug–matrix interactions [21]. These smaller pores can be fabricated using 20–40 V in sulphuric acid or 30–50 V in oxalic acid, providing actionable process parameters.

For dental implants requiring antimicrobial prophylaxis, intermediate release rates are appropriate to provide sustained protection during the initial weeks of osseointegration. Pore diameters of 50–75 nm, achievable through 40–60 V in oxalic acid, would provide release durations of 3–5 days for 50% release, which could be extended through deeper pores or multi-layer coatings [1].

Beyond these specific applications, the ML-diffusion modeling framework enables the systematic exploration of the design space to identify optimal anodization conditions for any desired release profile. This approach can dramatically reduce the experimental burden of implant development and potentially accelerate the translation of NAA-based drug delivery systems from the laboratory to the clinic.

An important consideration for practical implementation is the trade-off between the release rate and drug loading capacity. Larger pores provide faster release but also have greater loading capacity, as the drug reservoir volume scales with the pore diameter squared. For applications requiring both high loading and sustained release, this trade-off can be managed through hierarchical pore structures combining large pores for high loading with small pore entrances for controlled release [10], or through surface functionalization to slow release from large pores [21]. The ML framework developed herein could be extended to predict the properties of these more complex architectures as experimental data become available.

### 5.4. Comparison with Existing Approaches

The ML-based approach to NAA design presented herein represents a significant departure from traditional empirical methods and offers several advantages over existing approaches. Conventional NAA fabrication relies on trial-and-error experimentation guided by empirical rules of thumb, such as the 2.5 nm/V proportionality between voltage and interpore distance [15]. Although these rules provide useful starting points, they do not account for the complex interactions between multiple process parameters and often require extensive experimental optimization to achieve the desired pore characteristics.

Recent advances in the computational modeling of NAA formation have employed finite element methods and kinetic Monte Carlo simulations to predict pore morphology from first principles [2]. These physics-based models provide valuable mechanistic insights; however, they are computationally intensive and require detailed knowledge of material properties (e.g., oxide formation and dissolution rate constants), which are often not well characterized. Moreover, these models typically focus on idealized conditions and may not capture the full complexity of real experimental systems, including the effects of impurities, surface roughness, and nonuniform current distributions.

The ML approach offers a complementary middle ground between purely empirical methods and first-principles modeling. ML models implicitly capture the complex physics of NAA formation by learning from experimental data without requiring explicit mathematical formulations of the underlying mechanisms. This data-driven approach is particularly valuable for systems such as NAA, in which the governing equations are known qualitatively but are difficult to solve quantitatively because of coupled nonlinear processes. Rapid prediction times (<0.01 s per sample) enable real-time process optimization and high-throughput computational screening, which would be impractical with physics-based simulations.

In contrast to recent ML applications in drug delivery [22], the present work focuses on the synthesis–structure relationship rather than the structure–property relationship. Most ML studies in drug delivery predict release kinetics from known formulation properties (e.g., particle size, polymer composition), whereas we predict structural properties (pore diameter) from synthesis parameters (voltage, temperature, electrolyte). This synthesis-focused approach is more directly applicable to materials fabrication, as it provides explicit guidance on how to adjust process conditions to achieve the desired structure.

The integration of ML-predicted structures with physics-based release modeling (Section 4.5) represents a hybrid approach that combines the strengths of data-driven and mechanistic methods. The ML model handles the complex synthesis-structure relationship, in which first-principles modeling is challenging, whereas the Higuchi diffusion model handles the structure–property relationship, in which the physics is well understood. This hybrid strategy could serve as a template for ML applications in other material systems, in which some aspects are amenable to physics-based modeling, whereas others require data-driven approaches. To provide a more direct comparison with existing predictive approaches, the MLR model developed in this study was benchmarked against the widely cited empirical linear proportionality rule (pore diameter ≈ 1.29 × voltage for oxalic acid), which represents the most commonly used rule of thumb for NAA pore diameter estimation [2]. On the subset of the dataset corresponding to oxalic acid electrolyte (n = 18), the MLR model achieved an RMSE of 18.4 nm compared to 24.7 nm for the empirical proportionality rule, representing a 25% improvement. This demonstrates that even the simpler ML model outperforms existing single-parameter empirical models by incorporating additional process variables (temperature, time, and multi-electrolyte encoding). The ANN provided similar accuracy on this subset (RMSE = 19.1 nm). These comparisons further support the value of the multivariate ML approach, even at the current dataset size.

### 5.5. Limitations and Future Directions

Several limitations of the current work should be acknowledged and addressed in future research. First, the dataset size (n = 99, n = 77 for training) is relatively small for neural network training, limiting the model’s ability to learn complex nonlinear relationships and generalize to conditions far from the training data. Expanding the dataset through systematic literature review, data-sharing initiatives, or active learning experiments would likely improve model performance and enable more sophisticated architectures. A target of 200–500 samples would be ideal for fully leveraging neural network capabilities while maintaining manageable data collection efforts.

Second, the dataset is heterogeneous in terms of experimental methods, measurement techniques, and reporting standards across different research groups and time periods. This heterogeneity introduces noise that may limit model accuracy. Pore diameter measurements obtained using SEM, TEM, and AFM may differ systematically owing to sample preparation effects and measurement artifacts; for example, SEM measurements may slightly underestimate true pore diameter due to sputtered coating deposition (typical Pt/Au coating thickness: 5–10 nm per wall), whilst AFM tip convolution can overestimate pore wall sharpness. These systematic biases are unquantified in the current dataset and represent a source of uncontrolled variability. Furthermore, two important anodization parameters—current density and electrolyte concentration—were excluded from the model due to insufficient data coverage. Current density was reported in fewer than 30% of the dataset entries and its values cannot be reliably inferred from voltage alone, given circuit-dependent variations across different experimental setups. Electrolyte concentration, whilst recorded, suffered from inconsistent unit reporting (wt%, vol%, M) and high missingness after standardization. The impact of excluding these variables on model accuracy is acknowledged; in particular, including current density may improve model performance, as it is closely related to oxide formation rate and pore dimensions. Collecting datasets with complete reporting of all parameters—particularly current density and concentration—is strongly recommended for future work. Future work could address this through careful data curation, weighting of high-quality measurements, or explicit modeling of measurement uncertainty.

Third, the current model predicts only the pore diameter, whereas other structural parameters (interpore distance, pore depth, and pore wall thickness) are also important for drug delivery applications. Although pore diameter and interpore distance are strongly correlated (Figure 9), enabling indirect estimation of interpore distance from diameter predictions, pore depth is independently controlled by anodization time and would benefit from separate modeling. Multi-output neural networks that simultaneously predict multiple structural parameters could provide more comprehensive design guidance.

Fourth, the drug release simulations (Section 4.5) employ simplified assumptions about drug diffusion, including constant diffusion coefficients, uniform drug loading, and ideal cylindrical pore geometry. Real NAA structures exhibit pore size distributions, surface roughness, and branching that affect release kinetics [1]. Drug–matrix interactions, which can significantly retard or accelerate release depending on the drug’s chemical properties, are not explicitly modeled. Future work could integrate more sophisticated release models that account for these complexities, potentially using ML to predict drug-specific release parameters from molecular descriptors. Most critically, experimental validation of the ML model predictions has not yet been performed and represents the most important direction for future work. The model currently provides computational guidance based on existing literature data, but its practical utility for implant fabrication can only be confirmed through targeted experiments in which anodization parameters are selected based on model predictions and the resulting pore diameters are measured independently. The authors propose a validation protocol involving: (i) selection of 10–15 anodization conditions spanning the full parameter space (voltage: 20–150 V; electrolyte: all three types; temperature: 5–25 °C), guided by model predictions; (ii) fabrication of NAA samples under these conditions; (iii) SEM characterization of pore diameter; and (iv) comparison of measured vs. predicted diameters to assess real-world model accuracy. This experimental validation campaign is planned as a direct follow-up to the present computational work.

Fifth, the current model does not account for electrolyte concentration, which is known to affect pore morphology but was inconsistently reported in the literature dataset. Including concentration as an input feature would improve model completeness and enable the optimization of this additional process parameter. Similarly, current density, pH, and stirring conditions could be incorporated as additional features when more comprehensive datasets become available.

Sixth, the model is trained on data from conventional two-step anodization processes and may not generalize to advanced fabrication methods, such as pulse, modulated voltage, or hierarchical anodization [10,16]. Extending the framework to these more complex processes would require collecting data on time-varying process parameters and developing sequence-based models (e.g., recurrent neural networks) that can handle temporal dependencies.

Several promising directions for future research emerge from this work.

1. Active learning for data-efficient model improvement: Rather than randomly collecting more data, active learning strategies can identify the most informative experiments to conduct next, focusing on regions of parameter space where the model is most uncertain or where predictions deviate most from physical expectations [14]. This approach can accelerate model improvement while minimizing experimental effort;
2. Physics-informed neural networks: Incorporating known physical constraints (e.g., the proportionality between voltage and interpore distance, and the conservation of charge) into the neural network architecture or loss function can improve generalization and reduce data requirements [12]. This hybrid approach combines the flexibility of ML with the reliability of physics-based modeling;
3. Multi-fidelity modeling: Combining experimental data with results from physics-based simulations can expand the effective dataset size and enable the exploration of conditions that have not yet been studied experimentally. Transfer learning from simulations to experiments can bootstrap model training when experimental data are scarce;
4. Uncertainty quantification: Developing probabilistic models (e.g., Bayesian neural networks, Gaussian process regression) that provide confidence intervals on predictions would enable risk-aware process optimization and help to identify when experimental validation is most needed [22];
5. Inverse design optimization: Using the trained model within an optimization framework to identify anodization parameters that maximize specific objectives (e.g., minimize the release time while maximizing the loading capacity) would enable automated process design. Gradient-based optimization or genetic algorithms could efficiently search the parameter space guided by model predictions;
6. Extension to other nanoporous materials: The ML framework developed herein could be adapted to other electrochemically fabricated nanoporous materials, such as titania, zirconia, and porous silicon nanotubes, which share similar fabrication principles but differ in specific electrochemical behaviors [16]. Transfer learning could leverage knowledge gained from NAAs to accelerate model development for these related systems;
7. Integration with implant design tools: Incorporating the ML-diffusion modeling framework into computer-aided design (CAD) software for medical implants would enable engineers to optimize implant coatings as part of the overall device design process, while simultaneously considering mechanical, biological, and drug delivery requirements.

## 6. Conclusions

This study demonstrates the successful application of machine learning to predict nanoporous anodic alumina (NAA) pore morphology based on anodization process parameters, with direct implications for the rational design of drug-releasing implant coatings. By compiling a comprehensive dataset of 99 experimental observations from three decades of literature and training artificial neural network and multiple linear regression models, we developed predictive tools that capture the complex relationships between voltage, temperature, time, electrolyte type, and resulting pore diameter.

The key findings and contributions of the study include:

1. Predictive model performance: Both ANN and MLR models achieved strong training set performance (R^2^ ≈ 0.80, RMSE ≈ 26 nm), with MLR demonstrating superior generalization in cross-validation (CV R^2^ = 0.729 vs. 0.471 for ANN). This result highlights the importance of model selection based on dataset size, with simpler models often preferable for moderate-sized datasets;
2. Feature importance insights: Voltage was identified as the dominant predictor of pore diameter (86.32% importance in MLR and 29.15% in ANN), followed by electrolyte type (7.20% MLR and 30.23% ANN). The more balanced importance distribution in the ANN suggests that the neural network captures parameter interactions that are not represented in the linear model, providing insights for future mechanistic studies.
3. Parametric design guidance: Systematic analysis of voltage, temperature, and electrolyte effects provides actionable guidance for NAA fabrication. The pore diameter increases approximately linearly with voltage (~1.0–1.5 nm/V), decreases modestly with temperature (~0.4 nm/°C), and varies systematically with electrolyte type (sulphuric acid: 15–30 nm, oxalic acid: 40–80 nm, and phosphoric acid: 100–200 nm at typical voltages).
4. Drug release predictions: Integration of ML-predicted pore structures with Higuchi diffusion modeling demonstrates that pore diameter can be tuned to control drug release kinetics over a wide range, with release durations varying from ~1 day to ~7 days for 50% release by adjusting the pore diameter from 150 nm to 30 nm. This tunability enables the matching of release profiles to specific therapeutic requirements.
5. Design framework: The combined ML-diffusion modeling framework provides a computational tool for rational implant coating design, enabling researchers to identify anodization parameters that achieve targeted pore dimensions and release profiles without extensive experimental trial-and-error. This approach can accelerate the development of NAA-based drug delivery systems for orthopedic, cardiovascular, and dental applications.
6. Dataset and methodology: The compiled dataset and validated modeling approach provide a foundation for future research, including expansion to larger datasets, incorporation of additional process parameters, extension to multi-output predictions of multiple structural properties, and adaptation to related nanoporous material systems.

With the continued growth of datasets through ongoing research and data-sharing initiatives, and as ML algorithms become more sophisticated, the predictive accuracy and scope of these models will improve. The framework developed herein can be extended to predict additional structural parameters (interpore distance, pore depth, and pore wall thickness), handle more complex fabrication processes (pulse anodization and modulated voltage), and optimize multi-objective design criteria (balancing loading capacity, release rate, mechanical properties, and biocompatibility). Ultimately, the integration of ML-guided materials design with advanced fabrication techniques and clinical translation efforts promises to accelerate the development of next-generation drug-releasing implants, thereby improving patient outcomes across diverse therapeutic applications.

## Figures and Tables

**Table 1:** Dataset summary statistics for anodization parameters and resulting NAA pore characteristics.

Parameter	Min	Max	Mean	Std. Dev.	N (Samples)
Anodization Voltage (V)	5	400	65.7	69.5	97
Anodization Temperature (°C)	0	30	11.9	8.8	85
Anodization Time (min)	0.4	9600	395.9	1124	87
Pore Diameter (nm)	10	320	68.1	60.8	99
Interpore Distance (nm)	26.5	1000	147.1	209.4	53
Pore Depth (μm)	0.1	45	6.2	9.4	24

Note: N indicates the number of valid data points available for each parameter.

**Table 2:** Model performance comparison for ANN and MLR models.

Model	R^2^	RMSE (nm)	MAE (nm)	CV R^2^ (Mean ± Std.)	Training Samples
ANN (64-32-16)	0.8034	25.83	17.05	0.471 ± 0.078	77
Multiple Linear Regression	0.8037	25.8	15.89	0.729 ± 0.083	77

Note: R^2^ = coefficient of determination; RMSE = root mean square error; MAE = mean absolute error; CV = cross-validation. ANN architecture: 4 input features, 3 hidden layers (64-32-16 neurons), 1 output.

**Table 3:** Detailed R^2^ scores for each fold in 5-fold cross-validation comparing ANN and MLR models.

Fold	ANN R^2^	MLR R^2^	Difference (ANN − MLR)
Fold 1	0.3539	0.764	−0.4101
Fold 2	0.5025	0.6644	−0.1619
Fold 3	0.5778	0.6615	−0.0837
Fold 4	0.5082	0.6784	−0.1702
Fold 5	0.4144	0.8783	−0.4639
Mean	0.4714	0.7293	−0.2580
Std. Dev.	0.0783	0.0834	-

**Table 4:** Relative importance of input features in predicting pore diameter for both ANN and MLR models.

Feature	ANN Importance (%)	MLR Importance (%)	Rank (ANN)	Rank (MLR)
Voltage	29.15	86.32	2	1
Temperature	22.94	1.5	3	4
Time	17.69	4.98	4	3
Electrolyte Type	30.23	7.2	1	2

**Table 5:** The detailed statistical summaries of the electrolyte-specific pore diameters from the experimental dataset.

Electrolyte Type	Count	Mean (nm)	Std. Dev. (nm)	Min (nm)	Max (nm)
H_3_PO_4_ (phosphoric acid)	5	104	55.5	60	200
H_2_C_2_O_4_ (oxalic acid)	18	46.7	23.5	15	104
H_2_SO_4_ (sulphuric acid)	18	23.3	5.4	15.8	34.2

Note: Only electrolyte types with ≥3 samples are included to ensure statistical reliability. Results show significant variation in pore diameter based on electrolyte chemistry, with phosphoric acid typically producing larger pores than sulphuric or oxalic acids.

**Table 6:** Simulated Drug Release Kinetics Parameters.

Pore Diameter (nm)	Release Constant k (h^−0.5^)	Time to 50% Release (h)	Time to 90% Release (h)	Initial Release Rate (%/h)
30	0.68	54.3	174.1	6.8
50	0.8	39.1	126.6	8
75	0.95	27.7	89.7	9.5
100	1.1	20.7	67	11
150	1.4	12.8	41.3	14

Note: Predicted drug release parameters based on Higuchi diffusion model for different pore diameters.

## Data Availability

The original contributions presented in this study are included in the article. The raw experimental data supporting the findings of this study were extracted from published literature sources cited in the reference list. The compiled dataset used for model training is not independently available as a separate repository deposit at this stage; however, the raw data underlying each data point can be accessed directly from the original publications cited in this manuscript. Readers wishing to replicate this study are directed to the referenced sources. The authors are willing to share the compiled data table upon reasonable request to the corresponding author. Further inquiries can be directed to the corresponding author.

## Abbreviations

The following abbreviations are used in this manuscript:

AFM	Atomic force microscopy
AI	Artificial intelligence
ANN	Artificial neural network
MAE	Mean absolute error
ML	Machine learning
MLR	Multiple linear regression
MOFs	Metal–organic frameworks
NAA	Nanoporous anodic alumina
ReLU	Rectified linear unit
RMSE	Root mean square error
SEM	Scanning electron microscopy
TEM	Transmission electron microscopy
